# Psychodynamic-oriented Psychoeducation for Early-Stage Breast Cancer Patients: Effects on Clinical and Psychological Outcomes

**DOI:** 10.1192/j.eurpsy.2025.1780

**Published:** 2025-08-26

**Authors:** S. Jang, K. Kim, H.-D. Rim, J. Woo

**Affiliations:** 1 Psychiatry, Daugu Catholic University; 2 Psychiatry, Kyungpook National University, Daegu, Korea, Republic Of

## Abstract

**Introduction:**

Breast cancer is one of the most common cancers in women. Depression and anxiety affect not only health behaviors and treatment adherence but also cancer recurrence. Several studies have demonstrated that psychosocial interventions improve both mood symptoms and survival in breast cancer patients.

**Objectives:**

Psychodynamics plays a significant role in psychological distress and maladaptive behaviors. Therefore, psychoeducation focusing on patient dynamics can offer more individualized and tailored intervention. This study investigated the impact of psychodynamic-oriented psychoeducation for early-stage breast cancer patients on both psychological distress and disease trajectory, compared to those receiving standard cancer treatment only.

**Methods:**

Psychodynamic-oriented psychoeducation aims to provide guidance on managing psychological distress during cancer treatment, based on clinical and psychodynamic assessments of patients. A trained psychiatrist delivered this 60-minute intervention at least once within one-week post-mastectomy. The study included early-stage breast cancer patients (AJCC stages 0-IIIA) who underwent mastectomy at Kyungpook National University Hospital (KNUH) and Kyungpook National University Chilgok Hospital (KNUCH) between 2008 and 2015, excluding those with prior cancer history. Participants were divided into two groups: control (standard treatment) and treatment (standard treatment plus psychodynamic-oriented psychoeducation). Outcomes measured included breast cancer recurrence, disease-free survival, and psychological assessments using the Hospital Anxiety and Depression Scale (HADS) and Experiences in Close Relationships-Modified 36 (ECR-M36) at baseline and 12 months post-intervention in the treatment group. Propensity score matching was used to control for recurrence-related factors.

**Results:**

The median follow-up was 72.6 months. Recurrence rates were comparable between control and treatment groups (control vs. treatment: 9.0% vs. 8.3%, p=0.763). In a subanalysis of recurrent cases, the treatment group showed longer disease-free survival (51.3 vs. 32.5 months, p=0.038). (Table 1 and Figure 1) HADS scores showed no significant difference at 12 months, while ECR-M36 showed significant decreases in total and anxiety subscale scores at 12 months (p<0.05). (Table 2)

**Image:**

**Figure:**
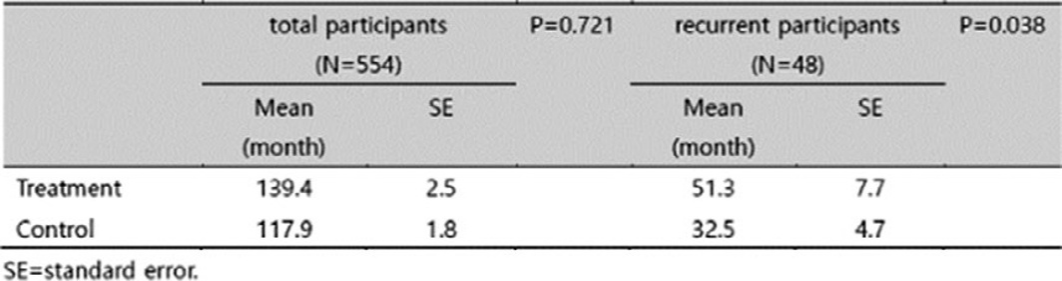

**Image 2:**

**Figure:**
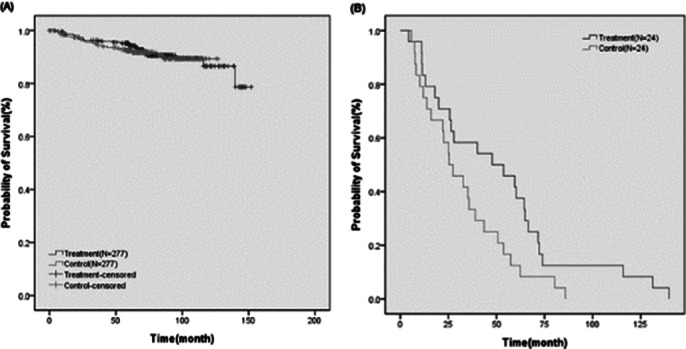

**Image 3:**

**Figure:**
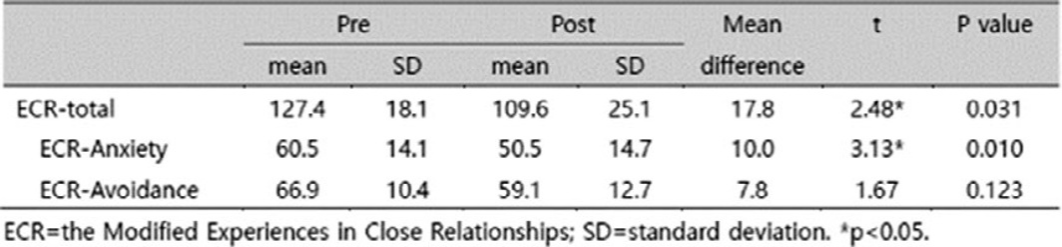

**Conclusions:**

This study, unique in integrating psychodynamic principles into psychoeducation, showed improvements in attachment-related anxiety and longer disease-free survival, specifically among recurrent cases, suggesting the potential benefits of this approach for certain breast cancer patients. Further research is needed to identify which patients might benefit most from this intervention.

**Disclosure of Interest:**

None Declared

